# Fast deliberation is related to unconditional behaviour in iterated Prisoners’ Dilemma experiments

**DOI:** 10.1038/s41598-022-24849-4

**Published:** 2022-11-24

**Authors:** Eladio Montero-Porras, Tom Lenaerts, Riccardo Gallotti, Jelena Grujic

**Affiliations:** 1 AI Lab, Vrije Universiteit Brussel, Brussels, Belgium; 2 MLG, Université Libre de Bruxelles, Brussels, Belgium; 3 Center for Human-Compatible AI, UC Berkeley, Berkeley, 94702 USA; 4 FARI Institute, Université Libre de Bruxelles-Vrije Universiteit Brussel, 1050 Brussels, Belgium; 5 Fondazione Bruno Kessler, Trento, Italy

**Keywords:** Human behaviour, Social behaviour

## Abstract

People have different preferences for what they allocate for themselves and what they allocate to others in social dilemmas. These differences result from contextual reasons, intrinsic values, and social expectations. What is still an area of debate is whether these differences can be estimated from differences in each individual’s deliberation process. In this work, we analyse the participants’ reaction times in three different experiments of the Iterated Prisoner’s Dilemma with the Drift Diffusion Model, which links response times to the perceived difficulty of the decision task, the rate of accumulation of information (deliberation), and the intuitive attitudes towards the choices. The correlation between these results and the attitude of the participants towards the allocation of resources is then determined. We observe that individuals who allocated resources equally are correlated with more deliberation than highly cooperative or highly defective participants, who accumulate evidence more quickly to reach a decision. Also, the evidence collection is faster in fixed neighbour settings than in shuffled ones. Consequently, fast decisions do not distinguish cooperators from defectors in these experiments, but appear to separate those that are more reactive to the behaviour of others from those that act categorically.

## Introduction

Experiments have shown that people differ in their motivations to cooperate (or not) in social settings^1–3^ and that these differences are shaped by, on the one hand, their inherent characteristics such as gender and age^4,5^ and, on the other hand, the context they find themselves in, such as the presence of certain social norms or institutions^6–8^, their network of contacts^9,10^ and repetition of those interactions^11,12^, among many other factors. It has furthermore been shown that people have different perceptions of what is fair and what is not: people may be inequality averse, hence interested in the alignment between the others’ actions and their payoffs with respect to their own^1,13^, while fairness differs also in how people value social welfare and concerns for efficiency in resource distribution^14^.

The details on why these differences appear among humans and what they mean, remain an area of active research. One mechanism proposed by researchers to describe individual heterogeneity in cooperation is Social Value Orientation (SVO)^15,16^, also known as other-regarding preferences^2,17^ or interdependent preferences for reciprocity^18^, among other names^19^. What these key concepts aim to measure is how humans perceive the importance of their own gains in relation to what others receive^1,20^. This way, there may be individuals that strongly think in terms of others, require the group as a whole to benefit, or prefer equality^1,19^. Some others might prefer to receive more than their opponents or even out-compete them, i.e. individualistic/competitive SVO or selfish preferences in the context of other-regarding preferences, or materialists in interdependent preferences.

These individual differences in cooperation have been associated with differences in the time people need to act or make a choice, i.e. their response time (RT). RT has, for instance, been analysed in the Ultimatum Game^21,22^ and the Prisoner’s Dilemma (PD) game^23^. Previous works on the relationship between RT and SVO specifically, focused on the Public Goods Game (PGG)^24,25^, Dictator Game, Ultimatum Game, and Trust Games (playing the role of the trustee)^26^, concluding that highly cooperative and highly individualistic participants are faster to decide. The explanation for this result was associated with the level of conflict a person perceives when making a decision^23,27–31^, rather than the competition between contrasting cognitive processes like deliberation or intuition^32^. Indeed, the use of behavioural or physical observations (such as RT) directly to explain mental processes remains unclear because this relationship might miss sources of variability in both the experiment and the data^29^.

Nonetheless, in the field of psychology, researchers have tried to understand and model the cognitive processes behind decision-making and RT for decades^33^. Progress has been made by linking neurosciences and behavioural economics^34–36^, which provides new insights into how different variables are related to the deliberation process in value-based decision-making, such as RT^37^. In traditional economic theory, it is assumed that people know exactly their preferences when choosing between two options when in reality, people deliberate over (noisy) subjective representations of these two options, which are encoded in neuronal firing rates^34^. One of the models that has tried to explain the relationship between the cognitive process of discrete-choice tasks and RT is called Drift Diffusion Model (DDM)^33,38,39^. DDM uses RT to model the cognitive processes of the participants in terms of initial bias (i.e. their preference for cooperation or defection), deliberation speed (i.e. how fast they collect evidence towards their preferred choice) and decision difficulty or carefulness^33^.

DDM has been used ever since in experimental settings in behavioural economics to study human deliberation processes and the psychological mechanisms of loss-aversion^40^, moral judgements^41^, inferring others’ preferences in binary decisions^42^, altruistic behaviour^43^ and food choices^44^, to cite a few. Mathematical decision models such as DDM have shown an accurate degree of generalization from one choice context to the other^44^. Furthermore, it was shown in a networked iterated PD (IPD) context how the participants’ perceived difficulty to make the decision decreased while their speed to collect evidence to make the decision increased at each iteration^45^. This work associated RT with the underlying cognitive process of deliberation and intuition, without making any assumptions about how fast the participants responded.

Here we show, through the use of DDM, that the cognitive processes of participants in a game-theoretical experiment are correlated to the heterogeneity in their predispositions and expectations to cooperate (or not). By linking the reaction times as modelled by the DDM with this heterogeneity, we are able to dig deeper into the individual cognitive differences in making decisions under different conditions. Specifically, the following two questions are addressed: (1) Are the predispositions to cooperate visible through the lens of DDM? (2) How do different decision contexts and game structures of the IPD play a role in our deliberation process? To answer these questions, we selected data from three different treatments with different network structures and payoff matrices, one where subjects play IPD in a pairwise setting, the second where they play the same game with multiple opponents in a network with a Moore’s neighbourhood setting (i.e., a square lattice with four direct neighbours), and the third experiment where participants were playing in a Von Neumann neighbourhood (i.e., a square lattice with eight direct neighbours). These three experiments were selected as they all have RT recorded, required the participants to make a binary choice and allow us to study the impact of the increasing interaction complexity on the RT of the participants. We inferred their cautiousness and speed to collect evidence throughout the games based on their DDM parameters. This way, their deliberation processes used to play these games are made explicit and can be associated with their individual behavioural heterogeneity.

As these experiments were not designed to directly collect data about individual SVO, other-regarding, or interdependent preferences; we propose here a measure that may serve as a proxy, which we will refer to as the Relative Allocation angle ($RA^{\circ }$) measure. $RA^{\circ }$ will represent the individual’s desired allocation towards self and others given the behaviour of their opponents in previous rounds (see “Methods”). $RA^{\circ }$ will allow us to examine the individual preferences in the IPD. This measure will assign each individual an angle: mostly cooperative will fall near $90^{\circ }$, mostly defective will fall around $0^{\circ }$, and conditional behaviour when the angle is close to $45^{\circ }$. We correlate these preferences with the cognitive parameters generated by the DDM model, allowing us to investigate whether knowing $RA^{\circ }$ accounts for insights into the cognitive processes of a participant. We offer an interpretation of the results and discuss how our results correlate with orthogonal neurological fMRI and eye-tracking studies on human cooperation.

## Results

### Participants reciprocated on pairwise settings, defected unconditionally in network games

**Figure 1:**
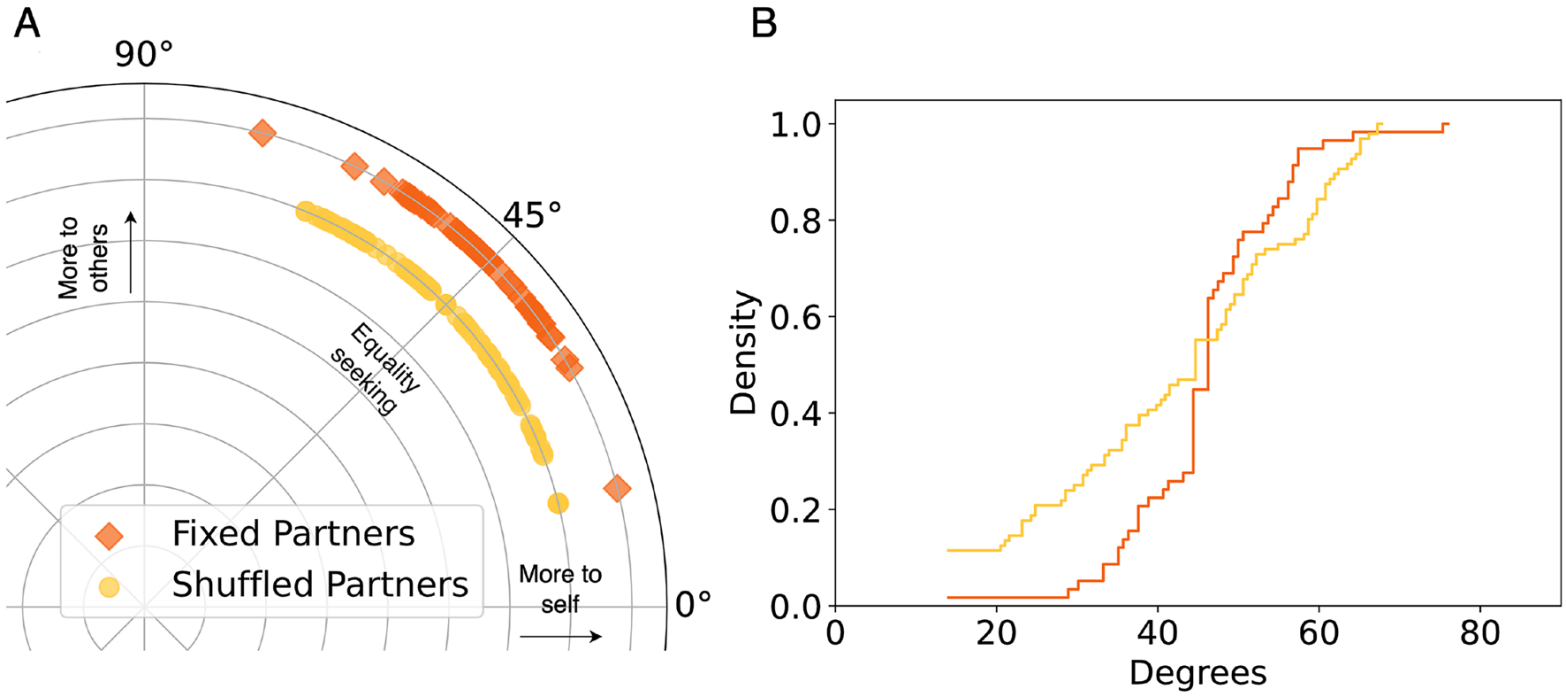
(**A**) Results of $RA^{\circ }$ in PIPD experiments^46^. Average payoff allocations, own vs. self. (**B**) Distribution of *RA* angles in the pairwise experiments. In PIPD$_f$ the vast majority resorted to reciprocate, resulting in an angle of $45^{\circ }$, 29 of the 57 participants (51%) ended up with a $RA^{\circ }$ between $40^{\circ }$ and $50^{\circ }$. In PIPD$_s$, 22 out of 96 participants (23%) were in this range.

**Figure 2:**
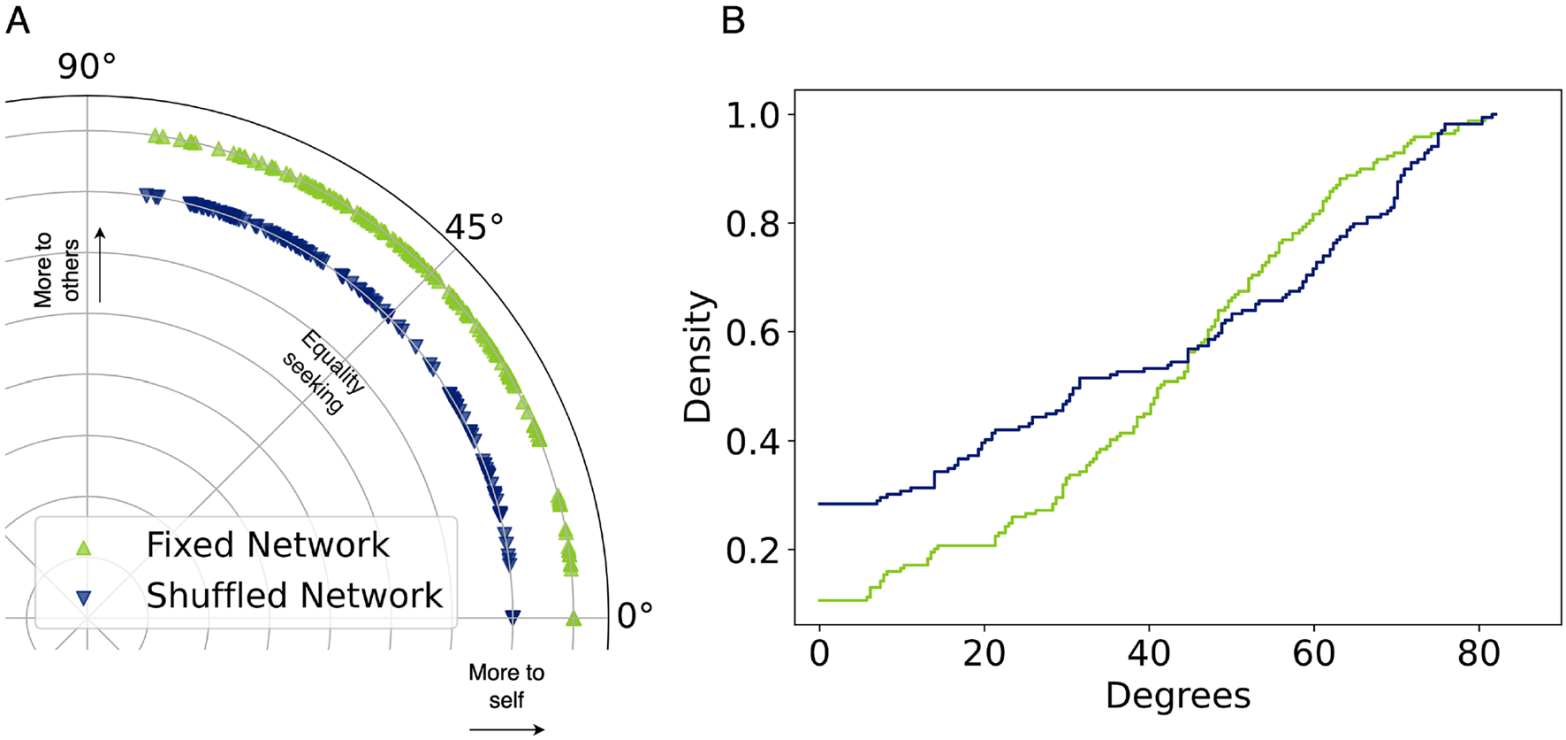
(**A**) Results of $RA^{\circ }$ in mNIPD experiments^47^. Average payoff allocations, own vs. self. (**B**) Distribution of RA angles in the mNIPD experiments. It is evident that many of the participants decided to either exploit their neighbours, resulting in a high concentration of participants with low levels of $RA^{\circ }$, in mNIPD$_f$ there were 27 participants with $RA^{\circ } <10^{\circ }$ (16%) and for the mNIPD$_s$ treatment, 52 had $RA^{\circ } < 10^{\circ }$ (31%).

**Figure 3:**
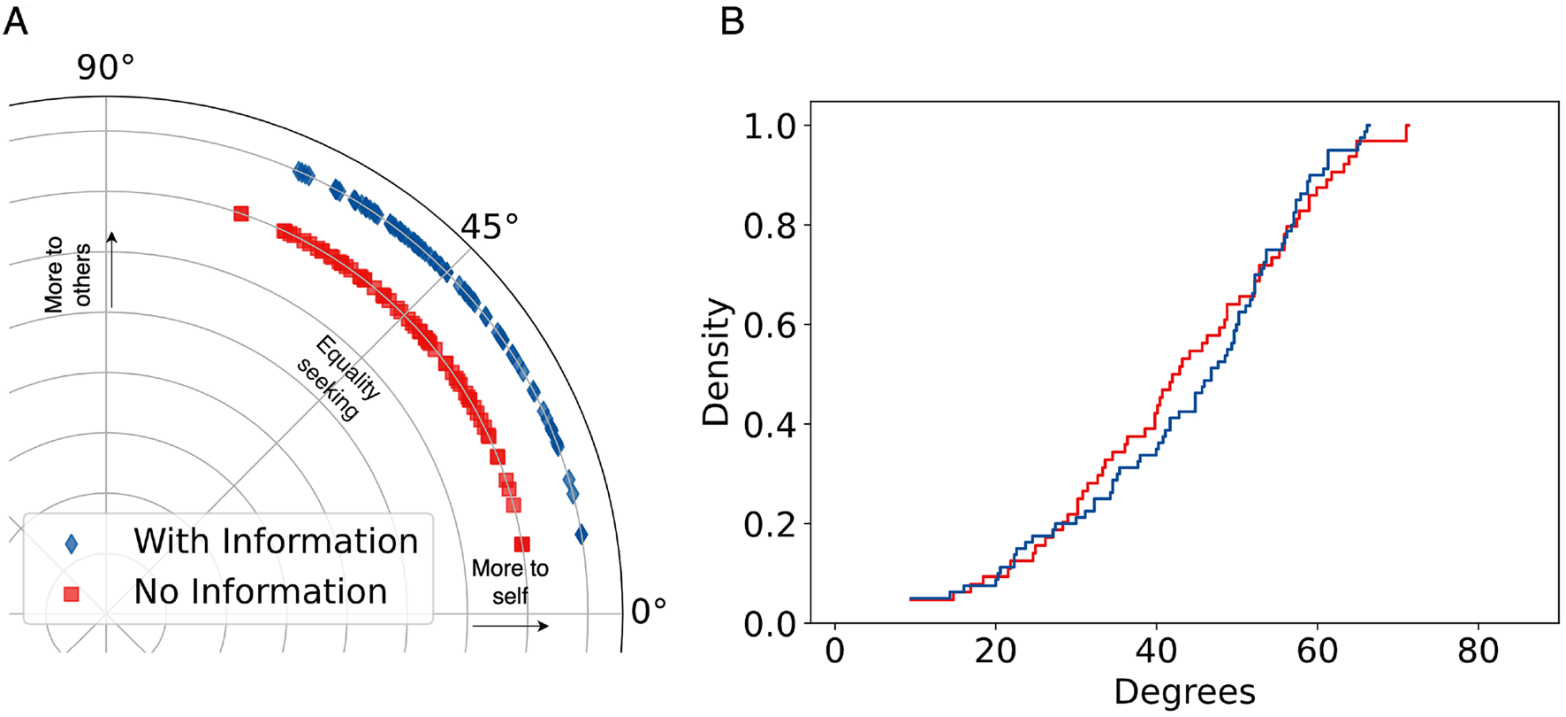
(**A**) Results of $RA^{\circ }$ in vnNIPD experiments^48^. Average payoff allocations, own vs. self. (**B**) Distribution of RA angles in the network experiments vnNIPD. The distribution of the angles does not look as skewed as in the mNIPD experiments, see Fig. 2, possibly due to the strong dilemma setting to make people reciprocate more and having less neighbours than the Moore Neighbourhood setting. Most participants were situated in the middle ($RA^{\circ }$ between $40^{\circ }{-}50^{\circ }$), 20 out of 80 participants (25%) in vnNIPD$_i$ and 15 out of 64 in vnNIPD$_o$ (23%).

Through the $RA^{\circ }$ measure, each participant’s preference towards their own gains relative to their co-players’ gains can be visualised, as shown in Figs. 1, 2 and 3. In the pairwise experiments (PIPD), the participants cluster around the $45^{\circ }$ degree mark in the fixed partners experiment, as the chart at the right shows. This distribution indicates that in fixed-partner setting (PIPD$_f$) subjects’ earnings appear to cluster around the equality marker. In shuffled partners (PIPD$_s$), the $RA^{\circ }$ distribution is spread out much more revealing a higher heterogeneity in allocations, this difference appears to be significant (KS statistic $D = 0.25$, p-value = 0.02). Nonetheless, in both cases, there appears to be a trend towards equal allocations between self and others.

In the network experiment mNIPD, the distribution in both the polar plane and the cumulative density plot, is shown in Fig. 2. There is a statistically significant difference (KS statistic $D = 0.21$, p-value = 0.001) between the distributions of the $RA^{\circ }$ scores in the fixed network setting mNIPD$_f$ and the shuffled network setting mNIPD$_s$, both with many participants defecting unconditionally leaving them at the bottom of the $RA^{\circ }$ distribution (near the zero degrees mark).

In the vnNIPD experiment, the density of $RA^{\circ }$ on the zero degrees mark (purely individualistic) appears to be 0. This may be due to the differences in the IPD payoff matrices, as was mentioned earlier (see Table 1). Both treatments in the vnNIPD experiments averaged $RA^{\circ }$ scores again near the equality marker. No significant difference is observed between the mean $RA^{\circ }$ scores of both treatments (KS statistic $D = 0.12$, p-value = 0.62). This means that while the subjects knew the payoffs of their opponents in the vnNIPD$_i$ treatment, the knowledge of their actions was enough to make their decisions as discussed in^48^.

### Evidence collection and perceived difficulty correlates with relative allocation

Now to examine the correlation between a participant’s deliberation process and an individual’s social preferences, we fitted the parameters *a*, *v* and *z* (threshold of the decision, drift speed and decision bias respectively) of the subjects against their $RA^{\circ }$ angle. The next sections detail the results for each of these parameters.

#### Decision bias: intuitive position before deliberation matters more in pairwise experiments

**Figure 4:**
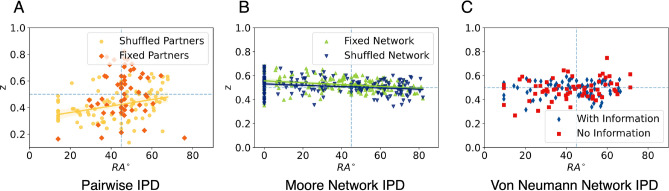
Average decision bias by their $RA^{\circ }$ per treatment. The decision bias *z* shows the initial point where the deliberation process between two options starts, the horizontal dotted line at $z = 0.5$ represents a point with no bias towards any option. It can be seen that in PIPD$_s$ and the vnNIPD experiments this relationship is positive, while in the mNIPD the bias and the $RA^{\circ }$ score follows a negative correlation. The lines across represent the linear regression and the shadows the 95% confidence interval. Note that in Panel A and C no significant correlation was observed in PIPD$_f$ (fixed partners IPD) and vnNIPD (Von Neumann network IPD), which is why no lines were drawn. The vertical dotted line represents $RA^{\circ } = 45^{\circ }$.

In the pairwise experiments, as shown in Fig. 4A, there is only a significant relationship between the decision bias and subjects’ $RA^{\circ }$ in the PIPD$_s$ (Spearman’s $\rho = 0.49$, p-value $< 0.001$), meaning that when the starting point of the deliberation process was lower than 0.5, the $RA^{\circ }$ is also small, while the opposite happens with subjects with high $RA^{\circ }$, resulting in a higher decision bias. This is the relation one would expect, suggesting that here players that intuitively tend towards cooperation would be expected to also allocate more resources to others. However, the same observation can not be made for the fixed network experiment mNIPD$_f$, where there is here a significant anti-correlation with decision bias (Spearman’s $\rho = -\,0.24$, p-value $= 0.001$), see Fig. 4B. A similar relationship was found in the mNIPD$_s$ treatment (Spearman’s $\rho = -\,0.22$, p-value $= 0.004$). This means that some of those with low $RA^{\circ }$ had a decision bias higher than 0.5, nearer to the cooperation boundary, but ended up defecting. This might be because, despite the initial intuition of each individual to cooperate, they had to deliberate more since they were in a setting with more opponents.

In the vnNIPD experiments, as shown in Fig. 4C, no significant correlation was found between the $RA^{\circ }$ and their decision bias in the vnNIPD$_i$ (Spearman’s $\rho = 0.12$, p-value $= 0.91$). Same result for the vnNIPD$_o$ treatment (Spearman’s $\rho = 0.19$, p-value $= 0.12$).

#### Decision threshold: more individualistic participants consider the game as more difficult

**Figure 5:**
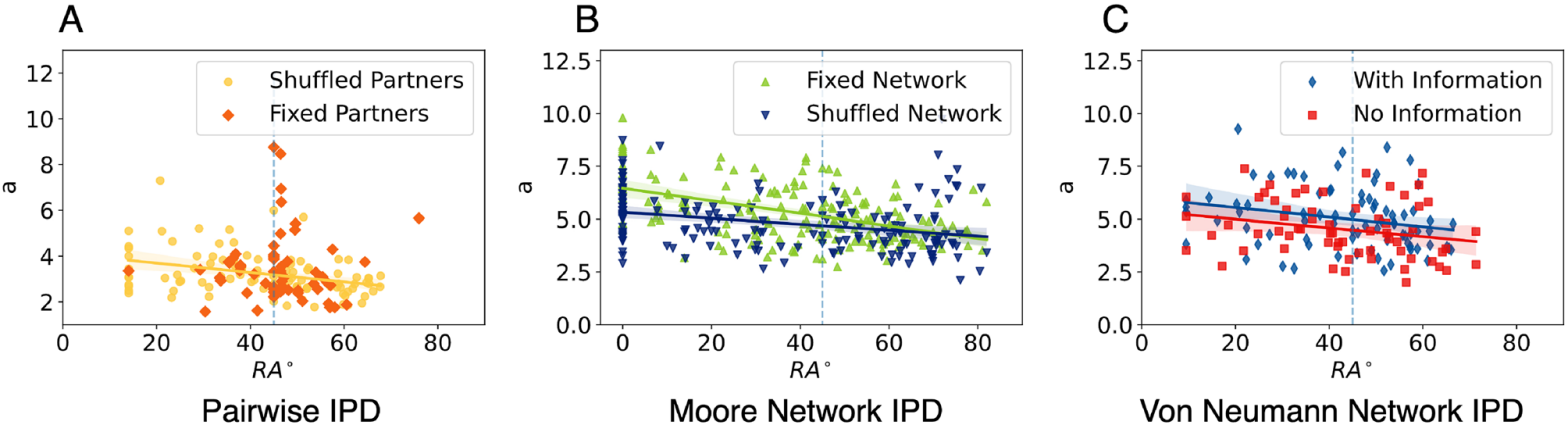
Relationship between the average decision threshold and their $RA^{\circ }$. The decision threshold *a* shows how wide the decision boundaries between two options are from each other, this can be interpreted as the perceived difficulty of the decision. It can be seen a significant negative correlation between the $RA^{\circ }$ score and their decision threshold, meaning that participants with low $RA^{\circ }$ scores found the settings more difficult. The diagonal lines represent the linear regression and the shadows the 95% confidence interval. The vertical dotted line represents $RA^{\circ } = 45^{\circ }$. Note that in Panel A no significant correlation was observed in the fixed partners IPD, (PIPD$_f$), which is why no orange line is drawn.

As shown in Fig. 5A, there is a significant negative relationship between the $RA^{\circ }$ and their threshold in the PIPD$_s$ experiment (Spearman’s $\rho = -\,0.40$, p-value $< 0.001$), meaning that for more selfish subjects the experiment was perceived as more difficult, as they tend to be more careful with their decision, gathering more evidence before committing to a decision, as their $RA^{\circ }$ decreased. In PIPD$_f$, subjects around $45^{\circ }$ experienced the experiments in all ranges of difficulty, while those with low or high values of $RA^{\circ }$ stayed around the same, no significant correlation between the threshold and $RA^{\circ }$ was found in PIPD$_f$ (Spearman’s $\rho = -\,0.15$, p-value $=0.29$).

In the mNIPD experiments, there is a significant negative relationship between $RA^{\circ }$ and their decision threshold in the mNIPD$_f$ (Spearman’s $\rho = -\,0.40$, p-value $< 0.001$) and mNIPD$_s$ (Spearman’s $\rho = -\,0.40$, p-value $< 0.001$), see Fig. 5B. This means that the subjects with low $RA^{\circ }$ (tendency for individualism) also experienced the experiments as more difficult than those with more cooperative tendencies.

Moreover, there is a significant difference between the decision threshold between the two treatments in mNIPD, where mNIPD$_f$ has a bigger threshold compared to mNIPD$_s$ (KS statistic $D = 0.17$, p-value = 0.02). This difference could be because participants played in mNIPD$_f$ first in a fixed network setting, and then they were put in a shuffled network afterwards for the mNIPD$_s$ treatment. It has been shown that participants use the first rounds to explore and learn the game^46^, hence increasing the perceived difficulty. No other significant differences in decision threshold were found in other experiments.

The vnNIPD experiments (see Fig. 5C) showed as well a negative relationship between the decision threshold and their $RA^{\circ }$. Although, the treatment with no information about opponents’ payoffs vnNIPD$_n$ showed a negative correlation with the decision threshold (Spearman’s $\rho = -\,0.24$, p-value $= 0.03$) and a stronger one in the treatment with information vnNIPD$_i$ (Spearman’s $\rho = -\,0.30$, p-value $= 0.006$).

#### Drift speed: fast evidence recollection is related to unconditional behaviour

**Figure 6:**
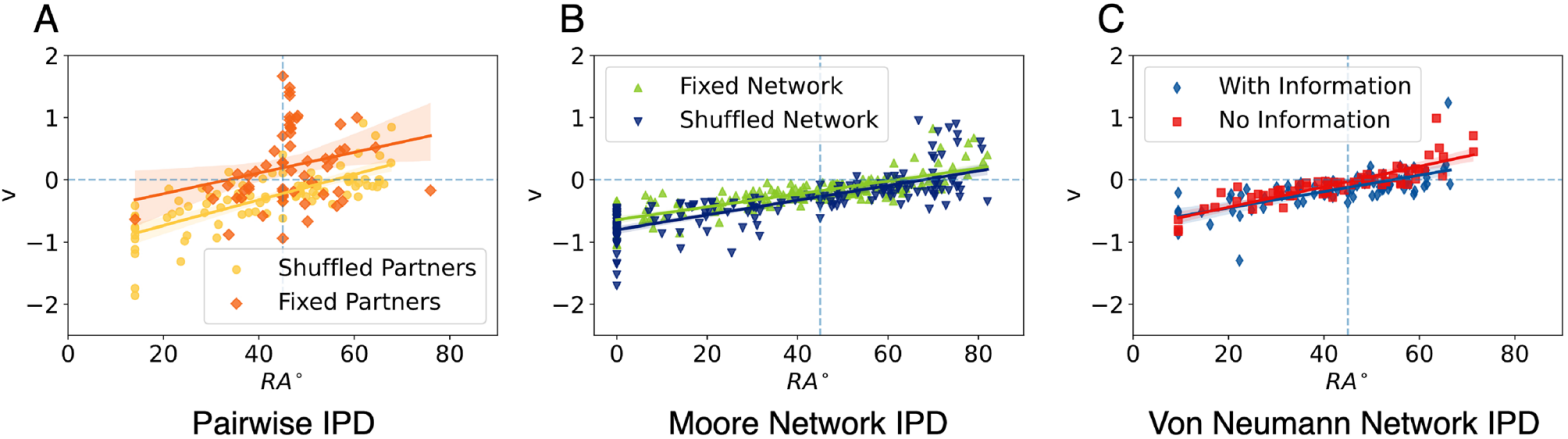
Relationship between the average drift speed and their $RA^{\circ }$ per treatment. The drift speed measures how fast the subjects accumulate evidence towards one of the two options, formatted as negative to defection and positive towards cooperation. The horizontal dotted line represents $v = 0$, or a point of no drift. It can be seen how subjects at both extremes of the $RA^{\circ }$ distribution accumulated speed faster towards their preferred option, while subjects near $45^{\circ }$ were the slowest, i.e. near zero drift speed *v*. The diagonal lines represent the linear regression and the shadows the 95% confidence interval. The vertical dotted line represents $RA^{\circ } = 45^{\circ }$.

As shown in Fig. 6A, the drift speed in the PIPD$_s$ experiment followed a linear relationship with $RA^{\circ }$ (Spearman’s $\rho = 0.84$, p-value $< 0.001$), as well as a positive relationship was found in the PIPD$_f$ experiment (Spearman’s $\rho = 0.29$, p-value = 0.03).

A significant relationship it is also shown in Fig. 6B, where in both treatments of mNIPD there is a high correlation between their $RA^{\circ }$ and their drift speed (Spearman’s $\rho = 0.77$, p-value $<0.001$ for the mNIPD$_f$ and Spearman’s $\rho = 0.81$, p-value $<0.001$ for the mNIPD$_s$ experiment).

In Fig. 6C, a similar situation is shown. In the vnNIPD, the information treatment vnNIPD$_i$ showed a high positive correlation (Spearman’s $\rho = 0.69$, p-value $<0.001$) and the no-information treatment (Spearman’s $\rho = 0.85$, p-value $<0.001$).

Also, there is a significant difference in average drift speed (*v*) between treatments in both PIPD and mNIPD. In PIPD$_f$, there is a positive average *v* ($\mu = 0.21, \sigma = 0.61$) when the partner is always the same and a negative average *v* ($\mu = -\,0.28, \sigma = 0.44$) for PIPD$_s$, meaning that subjects in these treatments collected evidence and drifted towards a different direction, i.e. cooperation and defection, respectively (KS statistic $D = 0.43$, p-value $< 0.001$). In mNIPD, both experiments drift towards defection (negative average *v*), although in the last part of the experiment with a shuffled network ($\mu = -\,0.39, \sigma = 0.43$) with a significantly greater speed (KS statistic $D = 0.31$, p-value $< 0.001$) with respect to the first part of the experiment with a fixed network ($\mu = -\,0.24, \sigma = 0.28$). No other significant difference was found among the treatments in vnNIPD.

This shows how for those with either low or high scores of $RA^{\circ }$, the drift speed goes higher towards their preferred strategy: defection, represented by negative drift speed; or cooperation, positive drift speed. Moreover, subjects around $RA^{\circ } = 45^{\circ }$, or subjects who responded more conditionally to their past interactions, had their drift speed near zero. This means that the evidence recollection speed for participants closer to the equality marker is slower than those who are further from $RA^{\circ } = 45^{\circ }$, i.e. participants who mostly defect or mostly cooperate. Moreover, having a fixed partner or network resulted in faster evidence collection compared with having a random co-player or network of co-players.

Moreover, to validate the results in general, we fitted a linear regression model per treatment and DDM parameter. The details of these models can be found in Supplementary Table S2. In general, it can be seen that the drift speed *v* is the variable that is able to explain better the variance in $RA^{\circ }$, as it corresponds to a significant coefficient in the linear regression and the higher Adjusted R-Square measure (last column on the table). Likewise, the decision bias *z* was not a significant coefficient in some treatments such as PIPD$_f$ and vnNIPD$_i$. The decision threshold *a* was significant but less able to explain the variance in $RA^{\circ }$.

Lastly, in their work, Gallotti et al. developed a measure to calculate how much of a decision is influenced by this a-priori intuition (represented in the DDM with the parameter *z*), where zero represents a decision with no deliberation, solely based on intuition, and one a decision based solely on deliberation. This measure was referred to as “rationality” (*R*). In Supplementary Fig. S2 can be seen how there is a significant negative correlation between $RA^{\circ }$ and their rationality measure *R*, except for the fixed partners treatment PIPD$_f$. This result means that while there are some participants with high $RA^{\circ }$ with high rationality *R* scores (upper right corner of each figure), most of the individualistic participants relied more on their deliberation rather than on their intuition, which also aligns with the result in the previous section regarding decision threshold, where the individualistic participants perceived the task as more difficult.

## Discussion

Our findings show that individual preferences in resource allocation, provided by the $RA^{\circ }$ proxy, influence the way subjects arrive and make decisions in multiple contexts and game structures, demonstrating again that DDM offers a richer analysis of response times.

The DDM decision bias, i.e. the starting point in the decision process that reflects the predisposition of participants, reveals a significant difference between the two pairwise treatments, suggesting that participants that played in the fixed-partners setting on average were more predisposed to cooperate than those in the shuffled partners’ treatment. Moreover, a positive correlation between the average decision bias and their $RA^{\circ }$ score in shuffled pairwise experiments was found, which contrasts with the negative one in the network experiments. Moreover, in all experiments (except for PIPD$_f$) there is a significant negative correlation between $RA^{\circ }$ and a rationality score *R*. This could mean that individualistic behaviour (higher $RA^{\circ }$) was related with a more deliberative decision-making process, as opposed to rely on their intuitive position.

Concerning the decision threshold, which measures the carefulness and perceived difficulty of the decision, participants in the mNIPD experiment were more careful in the first treatment (mNIPD$_f$) than in the one that took place later in the game (mNIPD$_s$). This might be due to the so-called “learning phase” in iterated games, where the first rounds are used to explore and learn the game^46^. Furthermore, we found that in settings such as PIPD$_s$ or both of the network experiments NIPD, there is a significant negative correlation between their $RA^{\circ }$ and their decision threshold, meaning that the lower their $RA^{\circ }$, the more difficult they perceive the game. The decision threshold makes the decision boundaries wider, hence the decision could potentially take longer.

Finally, there is also a significant positive correlation between the $RA^{\circ }$ and the drift speed in the shuffled partners and network experiments. Those with more extreme strategies (unconditionally cooperative, all-C, or unconditionally defective, all-D) apparently accumulated evidence towards their preference much faster than those in the middle of the $RA^{\circ }$ spectrum, whose allocated gains are almost equal to those of the others, also accumulating evidence slower. Meaning that those with unconditional strategies have a stronger preference for those options, although in some experiments, those who defected found the decision to do so harder than those that cooperated unconditionally. Also, we found differences between treatments in PIPD and mNIPD. Specifically, those in the shuffled partners drifted faster towards defection, while the fixed-partners players drifted towards cooperation. In mNIPD, subjects in the shuffled network drifted faster at the beginning of the game, even though they started with a fixed network, they ended up with a stronger preference for cooperation.

As mentioned in the introduction, previous works have linked neural activity with social values using techniques like fMRI^49,50^. This time, it is possible to look at the same problem with different lens provided by the DDM analysis. According to Emonds et al. pro-self (individualistic) strategies are driven by calculation, which can be compared with our finding in the section related to the decision threshold: In both PIPD and mNIPD experiments the threshold was negatively correlated with their $RA^{\circ }$ scores, meaning that participants with low $RA^{\circ }$ had a higher difficulty in arriving at a decision.

Our work also agrees with Fiedler et al. when it comes to the relationship with the $RA^{\circ }$ and their evidence collection. In their work, eye movement was tracked to show how SVO affects subjects’ strategies. As shown in Fig. 6, where subjects who mostly-defected or mostly-cooperated, drift-speed was higher, meaning that evidence to arrive at a decision was accumulated faster^51^. In a similar work, Bieleke et al. tested information acquisition and its relationship with SVO and deliberation, where they showed that less selfish individuals gathered more information about others and their payoffs^52^, reaching to a similar conclusion to our work and Fiedler et al. Although we also found high evidence collection (absolute value of *v*) in individualistic participants, we nonetheless observed that they relied more on their rationality rather than their intuitive a-priori position than their non-selfish counterparts. Also, our results are related to Capraro et al., where they measured participants’ cognitive style with the Cognitive Reflection test, and showed that deliberation promoted individuals’ concern for social efficiency (getting the most payoff for the group overall) but also egalitarian motivations were linked to intuitive responses^53^.

It is evident that the structures and strategic nature of the IPD game might have affected their $RA^{\circ }$ scores. For example, in PIPD$_f$, only the drift speed showed a significant correlation with the $RA^{\circ }$, meaning that the fixed opponent setting was a stronger influence of RT than the decisions under different conditions. Nevertheless, in other settings such as PIPD$_s$ and the network experiments, there were significant correlations between their $RA^{\circ }$ scores and their DDM parameters. Our findings are also in line with the findings of Andrighetto et al. They found that response time and cooperation are moderated by their SVO, where highly pro-social were faster to cooperate and highly individualistic were faster to defect^25^, with the difference that our work offers a much richer use of reaction times as a proxy to study our deliberation process. Since our results correlate with other works that emerged on the subject of deliberation, RT and heterogeneity in cooperation, it suggests that those findings are valid and useful for future research in human cooperation.

For future work, it is important to test these hypotheses with other games and settings, and perhaps with more than two options as presented in the IPD. This is crucial since in real life more than one setup and more than two options are presented in the complexity of human interactions, however, it is still of value to start to apply these concepts to different games and types of agents, such as artificial agents that can react faster than any human could, enriching our understanding of how response times affect our own (and also others’) deliberation process.

One has to acknowledge that DDM is still a new technique to combine with Game Theory experiments, as far as our knowledge goes. Still, it presents inviting opportunities to analyse individual differences in decision-making: we can go from measuring reaction times to arguing about social values and deliberation processes, and any researcher that desires to look deeper into the underlying processes of deciding these games (arguably without the need of an fMRI machine or an Eye-Tracking sensor). Moreover, this work adds to the theory of how different we are and to what extent, apart from all the techniques reviewed here, we could compare how DDM can be useful for future research.

## Methods

### Experimental IPD data

The analysis performed in this work relies on three experimental datasets wherein participants played different setups of the IPD. In the first, participants joined in a pairwise IPD (PIPD)^46^ whereas in the second they played the IPD in a network setting in a Moore Neighbourhood (mNIPD) which is a square lattice with eight direct neighbours^47^. In the third experiment, participants played in a network with a Von Neumann neighbourhood setting (vnNIPD)^48^ which consists in a square lattice composed of four direct neighbours. We include different network topologies and IPD experiments with different payoff matrices to validate our hypotheses. Specific information related to the datasets used here is provided in Supplementary Table S1. All the data used in this article is publicly available, see “Data availability” section.

We refer to the first experiment in^46^ as fixed partners or PIPD$_f$ ($n = 58$) and the second as shuffled partners or PIPD$_s$ ($n = 96$) throughout the rest of the paper. Note that, from the mNIPD experiment^47^, we consider the first fixed treatment (mNIPD$_f$, $n = 169$) from now on) and the following shuffled treatment (mNIPD$_s$, $n=169$). The second fixed treatment was not included as it did not add anything substantial to the results shown here, as it was also a fixed network just as mNIPD$_f$. Lastly, in the third experiment^48^, participants played vnNIPD in two treatments: one where they were informed of their opponents’ payoffs (vnNIPD$_i$, $n = 50$) in the previous round and one where they were not informed (vnNIPD$_o$, $n = 59$) (although in all experiments used in this paper, subjects knew the outcome of their opponent(s) actions in the previous round).

An additional difference between both datasets is that in the PIPD and vnNIPD the payoffs at each round were produced by a “strong” IPD where the following relation holds: $T>R>P>S$ and $2R>T+S$ (see Table 1). In the mNIPD, as can be seen also in the Table 1, a weak dilemma was used, where the two formal relationships mentioned earlier no longer hold.

**Table 1:** Payoff matrices used in the three experiments. The top payoff matrix shows the payoff used in the PIPD. Participants were confronted with a strong PD where $T>R>P>S$ and $2R>T+S$. The middle payoff matrix used in the vnNIPD experiment is also a strong dilemma, while the bottom payoff matrix corresponds to a weak one, as both conditions $T>R>P>S$ and $2R>T+S$ are no longer satisfied.

PIPD	C	D
C	R = 3/3	S = 0/4
D	T = 4/0	P = 1/1

vnNIPD	C	D
C	R = 5/5	S = 0/6
D	T = 6/0	P = 1/1

mNIPD	C	D
C	R = 7/7	S = 0/10
D	T = 10/0	P = 0/0

### Defining a proxy for social preferences

To measure the resource allocation of individuals, we took the response of the participants given the outcome of the previous round (which was known by participants) and averaged this over multiple rounds. We call this measure Relative Allocation angle or $RA^{\circ }$. The assumption that is made here is that a person’s allocated gains (as defined by the payoffs) to self and others, captured by $RA^{\circ }$, is correlated to a person’s social preferences.

To measure $RA^{\circ }$, we use the subjects’ planned allocation for themselves ($a_{self}$), and for others ($a_{other}$), i.e. whether they cooperate or not, given the context *c* of the decision, which is defined here as the number of co-players that cooperated in the last round played. This way, if we visualize them in a Cartesian plane as in Supplementary Fig. S1A, subjects end up situated at an angle from the origin of the plane. To account for different payoff matrices, we normalized the payoffs in all matrices, preserving the proportion of the R, S, T and P parameters, but limiting the range from 0 to 1. For more information about the $RA^{\circ }$ measure, motivation and examples, see the Supplementary Information.

We took the first 20 rounds of each experiment to develop the $RA^{\circ }$ metric, in order to have enough occurrences of each context and to be able to measure the preferences of cooperation of the participants (the results remain robust for a higher and lower number of rounds). By taking the mean over these rounds of ${\bar{a}}_{other}$ and ${\bar{a}}_{self}$, we can visualize where subjects ended up in a Cartesian plane, as shown in Supplementary Fig. S1. Equation (1) generates the angle from the origin (hence the $RA^{\circ }$ notation). This approach is equivalent to what Murphy et al.’s Slider Measure for Social Value Orientation^16^.

1 $$\begin{aligned} RA^{\circ } = \arctan \left( \frac{{\bar{a}}_{other}}{{\bar{a}}_{self}} \right) \end{aligned}$$

In terms of the IPD payoffs (see Table 1), the higher $RA^{\circ }$, i.e. near $90^{\circ }$, the more cooperative this person was when being confronted with defectors, consequently allocating more for others than for themselves. Clearly, cooperating when being exposed to defectors leads to the biggest payoff advantage for the others over self. This thus corresponds to behaviour like “All-C”. A $RA^{\circ }$ near zero means that this person allocated more for themselves than for others, i.e. playing defect when being confronted with persistent cooperation. This behaviour can be considered “individualistic” and is similar to “All-D”. Those around $45^{\circ }$ acted conditionally on their opponents’ past behaviour, responding with defect or cooperate depending on the observed context, and their own intentions. The net effect of the conditional response leads to an equal allocation of payoffs to self and others.

### Deliberation process measure

The three experiments, PIPD, mNIPD and vnNIPD, all recorded the response times of the participants for each decision made in the experiment. This way, following the work of Gallotti et al.^45^, we use the DDM to extract insights into the cognitive efforts made by the participants. As shown in an abstract representation of the DDM in Supplementary Fig. S1B, the deliberation process between two options occurs as the accumulation of evidence (with drift-speed *v*) towards one of the two options (cooperation or defection in our case) until a certain threshold of decision *a* is reached. The deliberation process starts at a point *z* (representing the initial motivations), and there is a time $t_0$ where no deliberation occurs^54^.

In this study, we analyse the values of the parameters *(a, v, z)* of the DDM: *a* indicates how difficult the decision is perceived by the subjects. *v* measures how fast each subject accumulates evidence to make their decision. These two measures describe a person’s cognitive/deliberation process while *z* reflects their initial bias towards one action or the other, i.e. the intuitive position before the deliberation process occurs. The models were fitted using the hddm package for Python^54^. The output of these models is a distribution of each parameter (*a*, *v* and *z*) and we report on the average of these distributions.

To make sure these distributions are stable, these models were tested for convergence, as hddm provides different methods to test the stability of the parameters over the rounds. A lack of convergence would mean that the distribution of the parameters is noisy, and therefore, not useful for analysis. To assess the models’ convergence, we used the Gelman–Rubin ${\hat{R}}$ statistic as recommended by the package’s creators and drew 10,000 posterior samples, discarding the first 200 as also recommended^54^.

To test the differences between samples in both average $RA^{\circ }$ and DDM parameters, we use a two-sample Kolmogorov–Smirnoff (KS) test.

## Supplementary Information

Supplementary Information.

## Data Availability

Both the code and the data used in this work are publicly available on the Zenodo repository: https://zenodo.org/record/6868396.
