# Supplementary material for: Fast deliberation is related to unconditional behaviour in iterated Prisoners’ Dilemma experiments
Source: Sci Rep. 2022 Nov 24;12:20287. doi: 10.1038/s41598-022-24849-4 (PMC9700794; doi:10.1038/s41598-022-24849-4)
Supplement: Supplementary file 1 — Supplementary Information. [file 41598_2022_24849_MOESM1_ESM.pdf]

Supplementary Information for: Fast deliberation is related to unconditional behaviour in iterated Prisoners' Dilemma experiments

Eladio Montero-Porras[1,*], Tom Lenaerts[1,2,3,4], Riccardo Gallotti[5], and Jelena Grujic[1,2]

[1]AI Lab, Vrije Universiteit Brussel, Brussels, Belgium
[2]MLG, Université Libre de Bruxelles, Brussels, Belgium
[3]Center for Human-Compatible AI, UC Berkeley, Berkeley, 94702, USA
[4]FARI Institute, Université Libre de Bruxelles-Vrije Universiteit Brussel, 1050 Brussels, Belgium
[5]Fondazione Bruno Kessler, Trento, Italy

November 17, 2022

Contents

1 Methods **2**
1.1 Experimental data 2
1.2 Relative Allocation measure 3

1 Methods

1.1 Experimental data

Experiment	Co-Players	Inter-action	Rounds	Parti-cipants	Type IPD	Reference
$PIPD_f$	2	fixed	100	96	strong	[1]
$PIPD_s$	2	shuffled	100	58	strong	[1]
$vnNIPD_i$	4	fixed	50	80	strong	[2]
$vnNIPD_o$	4	fixed	59	64	strong	[2]
$mNIPD_f$	8	fixed	60	169	weak	[3]
$mNIPD_s$	8	shuffled	47	169	weak	[3]

Supplementary Table S1: Details concerning the datasets used in the current analysis. There is one pairwise experiments (PIPD) with two treatments, and two network experiments (Von Neumann and Moore neighbourhoods) with two treatments each.

1.2 Relative Allocation measure

In order to quantify the behavioural heterogeneity in preferences of cooperation from the data of the different IPD experiments, an adaptation of the Slider Measure of Murphy et al. [4] is constructed here. Murphy and colleagues propose to examine at least 6 items, called the primary slider items, wherein subjects have to decide how to allocate a resource between themselves and another person over a continuum of joint payoffs. Ahn et al. constructed a similar measure to analyse possible payoffs, the extent of greed and inequality aversion in participants of behavioural experiments [5].

As this slider information was not acquired from the participants in the PIPD, mNIPD or vnNIPD experiments, their heterogeneity in social motivations needs to be determined differently. We define here the *Relative Allocation* (RA) measure as the allocation of payoffs to self and others relative to the allocations one experiences from others. The assumption that is made here is that a person's cooperative or individualistic motivations captured by the RA° approach are correlated to behavioural decisions that a person takes in a given game context. Here, the context is defined by the actions and gains one observes of the others in previous rounds while playing the IPD. We are aware that IPD constitutes a *strategic* game, where not only a participants' values are displayed but also their strategic nature, as mentioned by Murphy and Ackerman [6]. Nonetheless, measuring the preferences and heterogeneity of motivations in a given context is worth considering, as the observed trajectory of decisions will be correlated with a person's values. For example, how some participants may choose to defect facing defection from their opponent in the previous round while others choose to "forgive", might be the result of their values and expectations [1].

To measure RA, we used the planned allocation for themselves (a_{self}), and for others (a_{other}) that subjects had when taking a decision, i.e. whether they cooperate or not, given the context c of the decision, which is defined here as the number of co-players that cooperated in the last round played. This way, if we visualize them in a Cartesian Plane as in Figure S1A, subjects end up situated at an angle from the origin of the plane. In pairwise experiments, the context c can be $c = 1$ or $c = 0$, but in network experiments it could go from $c \in \{0, 1, .., 4\}$ in the vnNIPD and $c \in \{0, 1, .., 8\}$ in the mNIPD experiments. The calculation of a_{self} and a_{other} is given by Equations 1 and 2, respectively. N is the number of partners playing with the subject in the game, so for pairwise experiments $N = 2$ and for the network experiments, vnNIPD and mNIPD, $N = 4$ and $N = 8$ respectively. The parameters R, S, T and P are the payoffs used in the different IPD experiments. To account for different payoff matrices and even negative ones, we normalized the payoffs in all matrices, preserving the proportion of the R, S, T and P parameters, but limiting the range from 0 to 1.

$$a_{self} = \{ \; C, (R * c) + (S * (N - c)) \qquad \text{D}, (T * c) + (P * (N - c)) \tag{1}$$

$$a_{other} = \{ \; C, (R * c) + (T * (N - c)) \qquad \text{D}, (S * c) + (P * (N - c)) \tag{2}$$

For example, in PIPD$_f$, if a subject knows their partner cooperated in the previous round, they might choose to reciprocate, planning an allocation of 3 for each ($a_{self} = 3$ and $a_{other} = 3$), meaning that allocates the same for herself and her opponent, see the orange point in Figure S1A to see its position on the Cartesian plane. On the other hand, if they choose the defect, it means they planned an allocation of 4 for themselves and 0 for their partner ($a_{self} = 4$ and $a_{other} = 0$, see red point in Figure S1A).

A similar decision is taken in the network experiments, where a subject sees that in the previous round c people cooperated, so they have to decide their allocation given the context of the previous round. Then, for example, if in the previous round only 2 out of 8 participants cooperated ($c = 2, N = 8$), if a subject chooses cooperation, that would yield $a_{self} = 14$; and $a_{other} = 74$, see the green point in Figure S1A to see where it falls in the Cartesian plane.

We took the first 20 rounds of each experiment to develop the RA° metric, in order to have enough occurrences of each context and to be able to measure the preferences of cooperation of the participants. By taking the mean over these rounds for these two measures (represented by $\bar{a}_{other}$ and $\bar{a}_{self}$), we can visualize where subjects ended up in a Cartesian plane as shown in Figure S1, where their motivation can be determined by the angle from the origin (hence the RA° notation), as the Equation 3, which is equivalent to what Murphy et al. show [4].

$$RA^\circ = \arctan\left(\frac{\bar{a}_{other}}{\bar{a}_{self}}\right) \tag{3}$$

This way, the higher RA°, near 90°, the more cooperative this person remains while being confronted with defectors, consequently allocating more for others than for themselves. A behaviour thus similar to "All-C". A RA° near zero means that this person can allocate more for themselves than for others, i.e. playing defect when being confronted with cooperation. This behaviour can be considered "individualistic" and is similar to "All-D". Those around 45° acted conditionally on their opponents' past behaviour, responding with defect or cooperate depending on the observed context, and their own intentions.

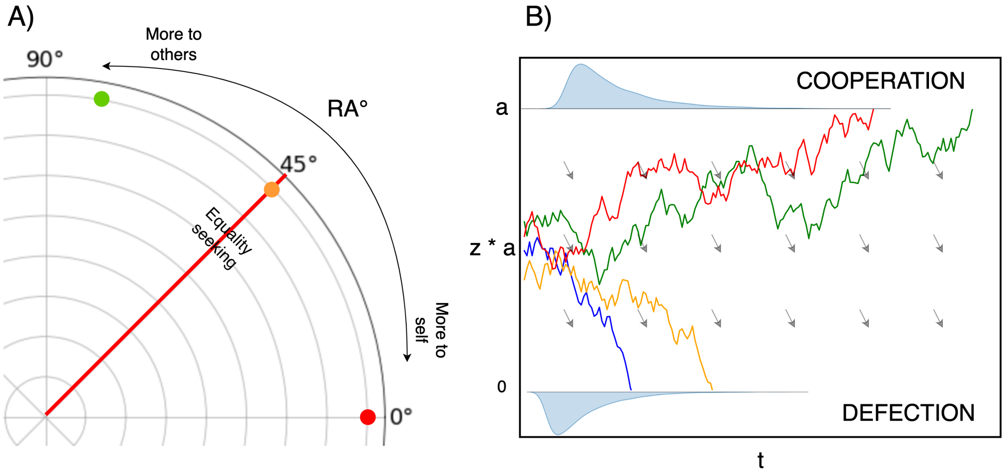

Supplementary Figure S1: Left: Visual representation of the Relative Allocation. The Relative Allocation (RA) is the ratio of how much each participant allocates for themselves (a_{self}) and for others (a_{others}. In this example, the points represent three different scenarios, the green point where a subject allocates 74 for others and 14 for herself in mNIPD, the orange point one where she allocates 3 for each in PIPD and the red one where a subject allocates zero for others and 4 for herself in the PIPD. See Methods for more details on this calculation. Right: Visual representation of the DDM as a series of random walks over time. The coloured lines represent a random walk of deliberation between two options: cooperation and defection. The parameter a represents the width of the decision spectrum, while z represents the starting point of the deliberation process, if it crosses this threshold, a decision is taken in time t. The arrows represent the drift that attracts the subjects towards defection in the IPD, and the blue curves the distribution of time t that subjects took to make the decision.

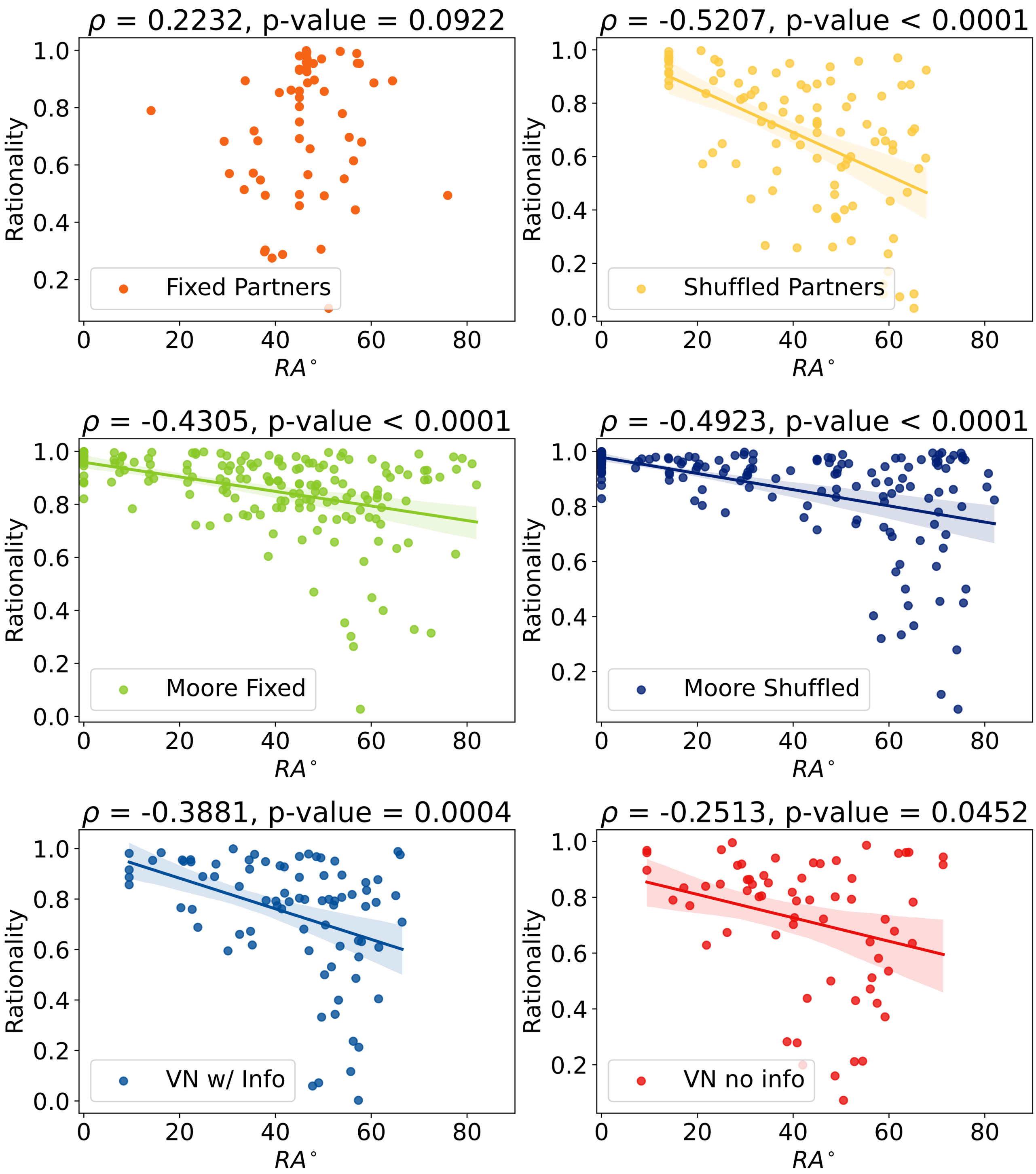

Supplementary Figure S2: Rationality measure vs. relative allocation per treatment. The rationality measure, developed by Gallotti et al. [7], measures how much each decision during the game is influences by the initial bias (z). Here, the scores were computed individually, each point represents a player, the lines indicate a significative Spearman correlation. It can be seen that except for $PIPD_f$ (upper right), all treatments present a negative correlation between RA° and their rationality measure. In other words, the more selfish the player was, the more they relied on deliberation, as opposed to players with high RA° that relied more on their intuition.

Experiment	Variable	Estimate	Std. Error	t-value	$Pr(>\|t\|)$	Significance	Adjusted R^2
PIPD$_f$	(Intercept)	43.018	3.912	11	1.31e-15	***	-0.004
	z	6.244	7.263	0.86	0.394		
	(Intercept)	45.8252	3.228	14.196	<2e-16	***	-0.01756
	a	0.1096	0.8566	0.128	0.899		
	(Intercept)	45.356	1.285	35.284	<2e-16	***	0.0505
	v	4.029	2.007	2.008	0.0495	*	
PIPD$_s$	(Intercept)	21.53	5.98	3.601	0.001	***	0.1117
	z	49.8	13.84	3.599	0.001	***	
	(Intercept)	61.087	5.485	11.14	<2.00e-16	***	0.11
	a	-5.799	1.625	-3.57	0.001	***	
	(Intercept)	49.861	1.306	38.17	<2e-16	***	0.55
	v	27.127	2.5	10.85	<2e-16	***	
mNIPD$_f$	(Intercept)	92.9	14.35	6.472	1.03e-09	***	0.07284
	z	-102.07	27.09	-3.768	2e-4	***	
	(Intercept)	81.183	6.063	13.39	<2e-16	***	0.23
	a	-7.933	1.109	-7.154	2.53e-11	***	
	(Intercept)	54.549	1.298	42.02	<2e-16	***	0.666
	v	64.277	3.506	18.33	<2e-16	***	
mNIPD$_s$	(Intercept)	85.97	16.61	5.174	6.50e-07	***	0.04891
	z	-100.03	32.22	-3.105	0.002	**	
	(Intercept)	67.408	8.105	8.316	3.05e-14	***	0.089
	a	-6.746	1.62	-4.163	5.01e-05	***	
	(Intercept)	55.19	1.857	29.71	<2e-16	***	0.611
	v	51.909	3.191	16.27	<2e-16	***	
vnNIPD$_i$	(Intercept)	38.477	13.316	2.89	0.005	**	-0.011
	z	9.646	26.864	0.359	0.721		
	(Intercept)	55.753	6.046	9.221	4.02e-14	***	0.044
	a	-2.497	1.158	-2.156	0.034	*	
	(Intercept)	48.579	1.398	34.739	<2e-16	***	0.465
	v	35.925	4.301	8.352	1.96e-12	***	
vnNIPD$_n$	(Intercept)	18.45	11.27	1.637	0.107		0.055
	z	48.9	22.69	2.155	0.035	*	
	(Intercept)	55.289	6.881	8.035	3.45e-11	***	0.042
	a	-2.847	1.456	-1.956	0.055	.	
	(Intercept)	45.74	1.062	43.06	<2e-16	***	0.728
	v	44.535	3.421	13.02	<2e-16	***	

Supplementary Table S2: Linear regression statistics for each of the DDM parameters with the RA° measure. The second to last column shows the significance in of each coefficient: < 0.001: '***', 0.001: '**', 0.01: '*' 0.05: '.' 0.1: ' '. The last column shows the adjusted R^2 for the linear model. In general, it can be seen that the parameter for the initial bias z is not significant in treatments such as vnNIPD$_f$ and PIPD$_f$. Plus, in all models, the drift speed v was the best parameter to explain the variance on RA°, compared to a and z.

References

1. Montero-Porras, E., Grujić, J., Fernández Domingos, E. & Lenaerts, T. Inferring strategies from observations in long iterated Prisoner's dilemma experiments. en. *Scientific Reports* **12.** Number: 1 Publisher: Nature Publishing Group, 7589. ISSN: 2045-2322. `https://www.nature.com/articles/s41598-022-11654-2` (2022) (May 2022).

2. Grujić, J. & Lenaerts, T. Do people imitate when making decisions? Evidence from a spatial Prisoner's Dilemma experiment. *Royal Society Open Science* **7.** Publisher: Royal Society, 200618. https://royalsocietypublishing.org/doi/full/10.1098/rsos.200618 (2021) (July 2020).

3. Grujić, J., Fosco, C., Araujo, L., Cuesta, J. A. & Sánchez, A. Social Experiments in the Mesoscale: Humans Playing a Spatial Prisoner's Dilemma. en. *PLOS ONE* **5.** tex.ids: grujicSocialExperimentsMesoscale2010a publisher: Public Library of Science, e13749. ISSN: 1932-6203. https://journals.plos.org/plosone/article?id=10.1371/journal.pone.0013749 (2020) (Nov. 2010).

4. Murphy, R. O., Ackermann, K. A. & Handgraaf, M. *Measuring Social Value Orientation* en. SSRN Scholarly Paper ID 1804189 (Social Science Research Network, Rochester, NY, Dec. 2011). https://papers.ssrn.com/abstract=1804189 (2020).

5. Ahn, T. K., Ostrom, E. & Walker, J. M. Heterogeneous Preferences and Collective Action. *Public Choice* **117.** Publisher: Springer, 295–314. ISSN: 0048-5829. https://www.jstor.org/stable/30025907 (2022) (2003).

6. Murphy, R. O. & Ackermann, K. A. Social Value Orientation: Theoretical and Measurement Issues in the Study of Social Preferences. en. *Personality and Social Psychology Review.* tex.ids: murphySocialValueOrientation2013a publisher: SAGE PublicationsSage CA: Los Angeles, CA. https://journals.sagepub.com/doi/10.1177/1088868313501745 (2020) (Sept. 2013).

7. Gallotti, R. & Grujić, J. A quantitative description of the transition between intuitive altruism and rational deliberation in iterated Prisoner's Dilemma experiments. en. *Scientific Reports* **9.** tex.ids: gallottiQuantitativeDescriptionTransition2019a, gallottiQuantitativeDescriptionTransition2019b, gallottiQuantitativeDescriptionTransition2019c number: 1 publisher: Nature Publishing Group, 1–11. ISSN: 2045-2322. https://www.nature.com/articles/s41598-019-52359-3 (2020) (Nov. 2019).
